# The DMPTool NIH DMSP Templates Project

**DOI:** 10.5195/jmla.2024.1871

**Published:** 2024-04-01

**Authors:** Nina Exner, Seonyoung Kim, Katy Smith

**Affiliations:** 1 nexner@vcu.edu, Research Data Librarian/Associate Professor, VCU Libraries, Virginia Commonwealth University, Richmond, VA; 2 seonyoung.kim@wustl.edu, Senior Support Scientist in Data Management and Sharing Services Group, Bernard Becker Medical Library, Washington University School of Medicine in St. Louis, St. Louis, MO; 3 katy.smith@slu.edu, Health Sciences Librarian, Medical Center Library, Saint Louis University, St. Louis, MO

**Keywords:** Research data management, Data management and sharing plans, DMPTool, NIH grant compliance, NIH data management and sharing plans

## Abstract

The DMPTool NIH Data Management and Sharing Plan (DMSP) Templates Project was launched in response to the 2023 NIH Data Management and Sharing (DMS) Policy. This new policy introduced a more structured framework for DMS Plans, featuring six key elements, a departure from the 2003 NIH DMS policy. The project aimed to simplify the process for data librarians, research administrators, and researchers by providing a template with curated guidance, eliminating the need to navigate various policies and guidelines. The template breaks out each Plan section and subsection and provides related guidance and examples at the point of need.

This effort has resulted in two NIH DMSP Templates. The first is a generic template (NIH-Default) for all ICs, complying with NOT-OD-21-013 and NOT-OD-22-198. More recently, an NIMH-specific template (NIH-NIMH) was added based on NOT-MH-23-100. As of October 2023, over 5,000 DMS Plans have been written using the main NIH-Default template and the NIH-NIMH alternative template.

## CONTEXT, AIMS, AND SIGNIFICANCE OF THE VIRTUAL PROJECT

When the National Institutes of Health (NIH) announced that expanded Data Management and Sharing (DMS) Plans would be part of the Final NIH Policy for Data Management and Sharing, effective January 25, 2023, data librarians wanted support. An existing virtual platform – the DMPTool – was already established as a source of templates for writing data management plans (DMPs) from other agencies.

DMPTool templates are constructed manually, so a new DMPTool NIH Template Working Group formed. The working group (WG hereafter) combined librarians with different strengths. Together this WG worked to synthesize and curate NIH DMSP templates as the 2023 NIH DMS Policy rolled out.

The main goals of this initiative were to:

1. Develop a generic NIH template that aligns with the NIH's Policy and optional Plan format.
2. Incorporate Genomic Data Sharing requirements, following NOT-OD-22-198, into the generic NIH Template.
3. Offer expanded example language across various disciplines, helping researchers understand how each element applies to their specific fields.
4. Evaluate the necessity for tailored templates for specific NIH Centers and Institutes (IC) and create IC-specific NIH DMS templates if deemed necessary.

The new 2023 NIH DMS policy affects multiple disciplines, from basic sciences to clinical and biobehavioral. The WG aimed to help researchers see how the new Plan format connects to their disciplines via example answers in the template. To support the increasing number of new medical centers joining the DMPTool, the WG also developed training materials (slide deck and flyers) and collaborated on a DMPTool training workshop for the Network of the National Library of Medicine.

## BRIEF DESCRIPTION OF THE VIRTUAL PROJECT

As the DMPTool was well-established before the NIH template project began, the NIH template goal was to use it to create a useful, standalone Plan template based upon the 2023 NIH DMS policy. The template provides not only writing prompts based on the NIH's Elements of a DMS Plan but incorporates other NIH documentation to support the writing prompts. Synthesized information plus example answers are labeled as “from DMPTool” to delineate them from NIH official text.

**Figure 1:**
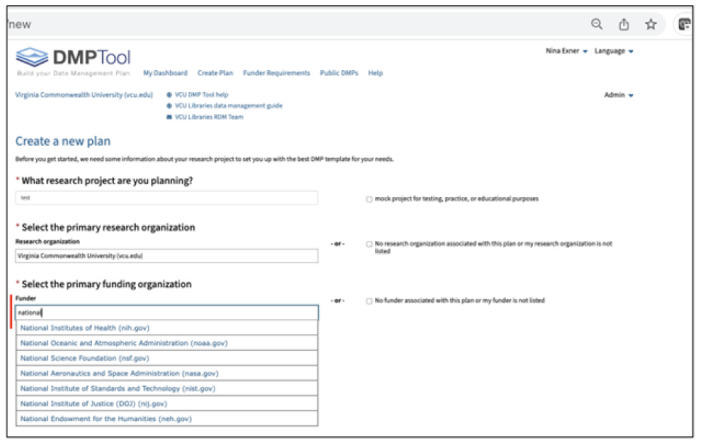


As shown in Figure 1, a user starts by selecting a funder to open the template tailored to that funder's guidance. For example, the template for writing DMPs for nutrition research funded by the USDA is different from that for nutrition research funded by the NIH. This project focused solely on a template for the NIH DMSP.

**Figure 2:**
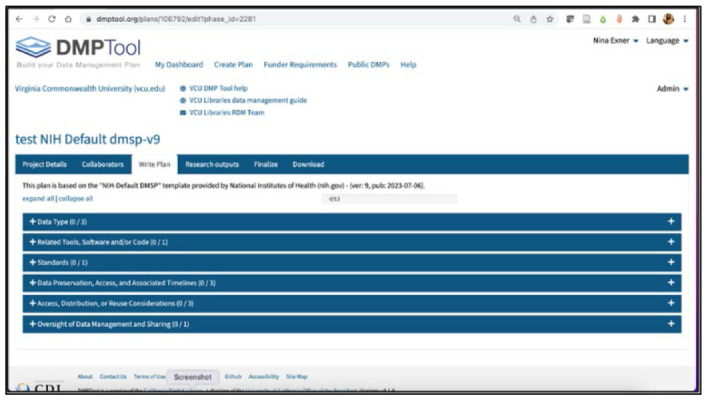


The user then encounters tabbed options. The “write plan” tab opens an accordion-menu-style screen with options specifically reflecting the NIH DMSP structure. This is where the value of the template becomes apparent: menus are based on the WG-created template. Figure 2 illustrates the menu of the main sections, with the sub-section menus closed.

**Figure 3:**
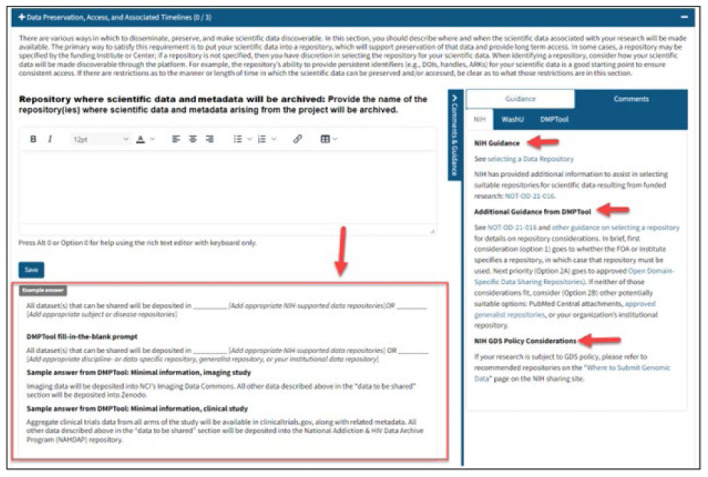


Clicking on one section bar opens the WG curated content. Each element or sub-element includes the official prompt from the Policy above the text-entry box followed by example answers below the text-entry box, with curated guidance in the right column under the NIH Guidance Tab. Figure 3 provides an example of the layout of Element 4 section.

The template has grown iteratively. In 2020, the DMPTool's Editorial Board created an initial 6-part template. Then a UC/Stanford-based team built the initial Example Answers version (v2), followed by the current WG version (v3), documented in an August 2022 DMPTool blog post. Evolutions such as integrating NIH sharing website content, harmonizing GDS policy considerations, and reflecting the optional Plan format (v4 - v9) were described in the January 2023 blog update. It has taken many librarians with various expertise to create the evolving product.

## TECHNOLOGY USED

The DMPTool is a free digital template platform provided by the California Digital Library (CDL). Anyone with an email can sign up to use it, at no cost. Institutions can become institutional participants if they work with the CDL/DMPTool team to integrate single sign-on and assign an administrator to run the institution-specific functions. The DMPTool also allows users to do collaborative authoring and route for feedback from the institutional DMPTool administrator(s) who are often data librarians.

## IMPACT

The primary impact is how many DMS Plans have been created using the templates we designed. Over 1,854 test or mock plans have been created from the NIH-Default template, plus 12 test plans from the NIH-NIMH template, which had been out for less than two months at count. Users can flag plans they create for test or mock-project uses like exploration or instruction.

Ultimately, the impact is in the non-test plans created. As of October 2023, DMPTool NIH Data Management and Sharing Plan (DMSP) Templates users have created 54 non-test plans with the NIH-NIMH template and 5,122 non-test-flagged plans with the main NIH-Default template.

## AUTHOR CONTRIBUTIONS

Nina Exner: conceptualization; writing – original draft; writing – review & editing; Seonyoung Kim: writing – original draft; writing – review & editing; Katy Smith: writing – original draft; writing – review & editing.

